# An algal lectin griffithsin inhibits Hantaan virus infection *in vitro* and *in vivo*

**DOI:** 10.3389/fcimb.2022.881083

**Published:** 2022-12-12

**Authors:** Yajing Zhao, Ningbo Zhao, Yanxing Cai, Hui Zhang, Jia Li, Jiaqi Liu, Chuantao Ye, Yuan Wang, Yamei Dang, Wanying Li, He Liu, Lianqing Zhang, Yuexiang Li, Liang Zhang, Linfeng Cheng, Yangchao Dong, Zhikai Xu, Yingfeng Lei, Lu Lu, Yingjuan Wang, Wei Ye, Fanglin Zhang

**Affiliations:** ^1^ College of Life Sciences, Northwest University, Xi’an, Shaanxi, China; ^2^ Department of Microbiology, School of Preclinical Medicine, Airforce Medical University, Xi’an, Shaanxi, China; ^3^ Guiyang Maternal and Child Health Care Hospital, Guiyang, Guizhou, China; ^4^ Key Laboratory of Medical Molecular Virology (MOE/NHC/CAMS), School of Basic Medical Sciences and BSL-3 Facility, Fudan University, Shanghai, China; ^5^ Department of Neurology, Xi’an International Medical Center Hospital, Xi’an, Shaanxi, China; ^6^ Department of Infectious Diseases, Tangdu Hospital, Airforce Medical University, Xi’an, Shaanxi, China; ^7^ Department of Pathogenic Biology, School of Preclinical Medicine, Yan’an University, Yan’an, Shaanxi, China

**Keywords:** griffithsin (GRFT), hantavirus, Hantaan virus, hemorrhagic fever with renal syndrome (HFRS), lectin, antivirals, suckling mice, vesicular stomatitis virus (VSV)

## Abstract

Hantaan virus (HTNV) is the etiological pathogen of hemorrhagic fever with renal syndrome in East Asia. There are currently no effective therapeutics approved for HTNV and other hantavirus infections. We found that griffithsin (GRFT), an algae-derived lectin with broad-spectrum antiviral activity against various enveloped viruses, can inhibit the growth and spread of HTNV. *In vitro* experiments using recombinant vesicular stomatitis virus (rVSV) with HTNV glycoproteins as a model revealed that the GRFT inhibited the entry of rVSV-HTNV-G into host cells. In addition, we demonstrated that GRFT prevented authentic HTNV infection *in vitro* by binding to the viral *N*-glycans. *In vivo* experiments showed that GRFT partially protected the suckling mice from death induced by intracranial exposure to HTNV. These results demonstrated that GRFT can be a promising agent for inhibiting HTNV infection.

## Introduction

Hantaan virus (HTNV) belongs to the family *Hantaviridae* and genus *Orthohantavirus* (hereinafter referred to as hantavirus). It causes hemorrhagic fever with renal syndrome (HFRS), which is a zoonosis endemic to East Asia, particularly in China (Jonsson et al., 2010; Jiang et al., 2017). It is transmitted to humans directly through inhalation of rodent excreta and bites or indirectly by consuming contaminated food (Jonsson et al., 2010). Acute HFRS symptoms, including fever, hemorrhage, and acute kidney injury, account for its high mortality rate of up to 15% (Jiang et al., 2016). As approved therapeutics for HFRS are currently lacking, there is an urgent need to develop new antiviral agents.

**Figure 1:**
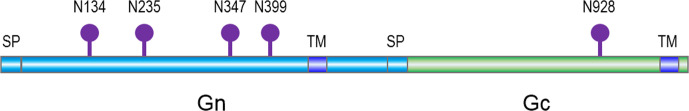
** **A schematic of Hantaan virus (HTNV) glycoprotein and predicted asparagine-linked glycosylation sites (lollipops), with signal peptide (SP) and transmembrane domains (TM).

HTNV has a tripartite genome consisting of L, M, and S segments encoding RNA-dependent RNA polymerase (RdRp/LP), glycoprotein precursor (GPC), and nucleocapsid protein (NP), respectively (Vaheri et al., 2013; Muyangwa et al., 2015). Upon maturation, GPC is processed into Gn and Gc, which form a tetrameric Gn/Gc spike embedded within the viral membrane envelope (Meier et al., 2021). Gn/Gc tetramer is crucial to initiating HTNV attachment and subsequent cell entry (Serris et al., 2020). Consequently, it represents a promising target for antiviral drug development. Recent structural studies have shown that putative *N*-linked glycosylation sites on both Gn and Gc (**Figure 1**) are pivotal to the proper folding of the Gn/Gc tetrameric complex (Serris et al., 2020). Griffithsin (GRFT) is a homodimeric lectin that binds high-mannose oligosaccharides, especially *N*-linked high-mannose oligosaccharides, found in viral glycoproteins (Lusvarghi and Bewley, 2016; Lee, 2019). GRFT has shown robust antiviral activity against various enveloped viruses, including coronavirus (O'Keefe et al., 2010; Li et al., 2019; Cai et al., 2020; Alsaidi et al., 2021), flavivirus (Meuleman et al., 2011; Ishag et al., 2013), and retrovirus (Mori et al., 2005), and is currently in phase I clinical trials against human immunodeficiency virus type 1 (HIV-1) (ClinicalTrials.gov Identifiers: NCT04032717, NCT02875119).

In this study, GRFT was found to exhibit promising inhibitory effects against HTNV infection. Furthermore, we discovered that GRFT inhibited the entry of recombinant vesicular stomatitis viruses (rVSVs) containing HTNV glycoproteins (rVSV-HTNV-G). Pretreatment with mannose blunted GRFT’s inhibitory activity against HTNV infection, supporting the hypothesis that GRFT’s glycan-binding property is essential for preventing HTNV infection. In addition, after mutating *N*-linked glycosylation sites, pseudoviruses carrying these mutations were packaged, and the infection caused by pseudoviruses with triple mutations (N347Q-N399Q-N928Q) was nearly unaffected by GRFT, demonstrating that GRFT blocks HTNV entry *via N*-glycans. Furthermore, in the suckling mouse model, GRFT partial protection against HTNV infection. Collectively, these results show that GRFT can inhibit viral replication by blocking HTNV entry, suggesting its potential therapeutic application in HTNV and other hantavirus infections.

## Materials and methods

### Cells, viruses, and reagents

African green monkey kidney (Vero E6) (CCL-81; ATCC, Manassas, VA, USA), human non-small-cell lung carcinoma (A549) (ATCC, CCL-185), Syrian golden hamster kidney (BHK-21) (ATCC; CCL-10), and human hepatoma (Huh7) cells were cultured in Dulbecco’s modified Eagle’s medium (DMEM; Sigma-Aldrich, St. Louis, MO, USA) supplemented with 10% fetal bovine serum (FBS; Sigma-Aldrich) in 5% CO_2_ at 37°C as described previously (Ye et al., 2020). HTNV (strain 76-118) was propagated and titrated in Vero-E6 cells as previously indicated (Ye et al., 2015; Ye et al., 2019). The primary mouse monoclonal antibody against HTNV NP (1A8) was produced in the lab, as mentioned previously (Ye et al., 2019). Antibodies against GAPDH and Alexa 488-conjugated goat-anti-mouse antibodies were acquired from Sangon Biotech (Shanghai, China). Horseradish peroxidase (HRP) was obtained from Sangon Biotech. Hoechst 33258 was purchased from MedChemExpress (Monmouth Junction, NJ, USA). GRFT was expressed and purified as previously described (Giomarelli et al., 2006; Cai et al., 2020). D-mannose was purchased from AbMole (Houston, TX, USA).

### Cytotoxicity assay

Cell viability was calculated as previously mentioned (Ye et al., 2020). Briefly, Vero-E6 and A549 cells were incubated with serial dilutions of GRFT for 72 h. Cell viability was assayed using Cell Counting Kit-8 (CCK8) (TargetMol, Shanghai, China), with absorbance (A) at 450 nm measured using a BioTek HT synergy instrument (BioTek, Winooski, VT, USA).

### Immunofluorescence image-based assay for antiviral activity

Cells were seeded in 48-well plates at a confluence of 60%–70%. To evaluate the inhibition of HTNV replication by GRFT, we treated viral stocks with the indicated concentration of GRFT at room temperature for 1 h. Then, cells were infected with GRFT-treated HTNV at a multiplicity of infection (MOI) of 1 for 2 h with rocking every 15 min. After absorption, the inoculum was removed, and the medium was replenished. At 72 h post-infection, the monolayers were fixed with 4% paraformaldehyde for 15 min at room temperature, then washed three times with Dulbecco’s phosphate-buffered saline (DPBS) and permeabilized with 0.5% (v/v) Triton X-100 in DPBS for 10 min. Then blocked with 3% bovine serum albumin (BSA) in DPBS, the plate was then incubated with hantaviral nucleoprotein-specific mouse monoclonal antibody 1A8 (diluted in DPBS supplemented with 3% BSA) at 4°C overnight as previously indicated (Ye et al., 2019). Alexa 488-conjugated goat-anti-mouse antibody served as a secondary antibody and was incubated at 37°C for 2 h. Cell nuclei were stained with Hoechst 33258, and the samples were imaged using an IX71 fluorescence microscope (Olympus, Tokyo, Japan). Five fields were collected for each group, and three independent experiments were performed. The fluorescence intensity of each image was quantified using ImageJ and normalized with positive control.

### Rescue of recombinant vesicular stomatitis virus bearing Hantaan virus glycoprotein precursor and recombinant vesicular stomatitis virus-based assay for antiviral activity

Plasmid-bearing VSV antigenome was synthesized at GenScript (Nanjing, China), containing unique restriction sites for foreign gene expression: 5′ flanked by the T7 bacteriophage promoter, 3′ flanked by hepatitis delta virus ribozyme (HDVRz), and the T7 terminator sequence. The codon-optimized GPC genes of HTNV (GenBank accession: NC_005219) were amplifled by PCR and inserted into the VSV antigenome plasmid, resulting in an rVSV-HTNV-G vector, and a green fluorescent protein (GFP) reporter gene was inserted after the HTNV GPC (Wang et al., 2022). The rVSV rescue follows the established protocol (Whelan et al., 1995). Briefly, BHK-21 cells were seeded into a six-well plate overnight and infected with vaccinia virus bearing T7-pol (kindly provided by Wuhan Institute of Virology, CAS) for 2 h. Then cells were transfected with helper plasmids encoding VSV-N, VSV-P, VSV-L, and VSV-G (kindly provided by Wuhan Institute of Virology, CAS). The transfection ratio for each plasmid is 5:3:5:1:8, with a total of 11 μg per well. Transfections were performed using Hieff Trans Liposomal Transfection Reagent (Yeasen, Shanghai, China). Cytarabine (TargetMol) was added after transfection at a concentration of 100 μg/ml, and culture supernatant was collected 72 h post-transfection and used to infect Vero E6 cells. The cytopathic effect (CPE) in the cell monolayer becomes evident after several passages, and the GFP expression indicates that the rescue was successful. The rescued virus was referred to as rVSV-HTNV-G and verified by HTNV GPC-specific antibodies. Viruses were propagated and titrated in Vero E6 cells, and titer was determined using plaque assays.

Cells were seeded in 48-well plates at a confluent of 60%–70%; after adhesion, the cells were infected with rVSV-HTNV-G. GRFT was applied or not applied to the viruses depending on the desired experiments. Then, cells were imaged with an IX71 fluorescence microscope 12 or 24 h post-infection. Five fields were collected for each group, the fluorescence intensity of each image was quantified using ImageJ and normalized, and three independent experiments were performed.

### Quantitative reverse transcription PCR

A549 cells were infected with HTNV (MOI = 1), which were treated with varying concentrations of GRFT for 1 h, and the inoculum was removed after virus adsorption and washed twice. After 72 h of treatment, cellular RNA was isolated and subjected to reverse transcription and subsequent qRT-PCR. Total cellular RNA was extracted using E.Z.N.A. Total RNA Kit I (OMEGA BioTek, Norcross, GA, USA). RNA was reverse transcripted with Hifair 1st Strand cDNA Synthesis SuperMix (Yeasen Biotechnology, Shanghai, China), and RNA concentration was determined with an Epoch microplate spectrophotometer (BioTek). Reverse transcription (RT) was then performed with a Hifair^®^ III 1st Strand cDNA Synthesis Kit (Yeasen) according to the manufacturer’s instructions. The cDNA was subjected to qRT-PCR performed using Hieff qPCR SYBR Green Master Mix (Yeasen). The mRNA expression level of each target gene was normalized to the corresponding GAPDH expression level. The primers used for gene amplification were qGAPDH-F: 5′-ACCCACTCCTCCACCTTTG; qGAPDH-R: 5′-ATCTTGTGCTCTTGCTGGG; qHTNV S-F: 5′-GAGCCTGGAGACCATCTG and qHTNV S-R: 5′-CGGGACGACAAAGGATGT.

### Western blotting analysis

Cells in six-well plates were treated and infected with HTNV as indicated. Cells were washed twice with DPBS and lysed with radioimmunoprecipitation assay buffer (RIPA buffer) (Beyotime, Shanghai, China) with protease inhibitor cocktail (MedChemExpress) and quantified using Pierce™ BCA Protein Assay Kit (Thermo Fisher Scientific, Waltham, MA, USA). Aliquots measuring 30 μg of each cell lysate were mixed with sample buffer supplemented with β-ME, boiled for 10 min, subjected to 12% sodium dodecyl sulfate–polyacrylamide gel electrophoresis, and transferred to polyvinylidene difluoride membranes (Merck Millipore). Membranes were incubated with antibody 1A8 to detect HTNV nucleoprotein, and glyceraldehyde 3-phosphate dehydrogenase (GAPDH, Sangon) was used as an internal control followed by secondary antibodies conjugated to HRP (Sangon). The membranes were visualized with the Tanon 5200 Chemiluminescent Imaging System (Tanon, Shanghai, China).

### *In vivo* evaluation of the antiviral activity of griffithsin against Hantaan virus infection

Specific-pathogen-free suckling mice (3 days old) were obtained from the Animal Research Center of Airforce Medical University. The acceptable infection dose was determined based on the 50% lethal dose (LD_50_) of HTNV intracranially (i.c.) inoculated in suckling mice. The mice were divided into three groups (one litter per group), and 10 LD_50_ of HTNV stock were incubated with GRFT with dosage regimens of 0 μg/ml (n = 17), 20 μg/ml (n = 11), and 100 μg/ml (n = 14) and were inoculated intracranially. Normal controls were inoculated with 2% DMEM (vehicle). All work with these animals was performed in the high containment of the Animal Biosafety Level Laboratory and was approved by the Institutional Animal Care and Use Committee. Mice were observed twice daily for mortality for 10 days after infection. Death occurring 48 h after viral inoculation was considered traumatic and excluded from the analysis. Log-rank test was performed to compare the significance of the survival curves.

### Hantaviral glycoprotein precursor pseudotyped vesicular stomatitis virus preparation and assays

GPC genes of HTNV (NC_005219), Seoul virus (SEOV, AB027521), Puumala virus (PUUV, U14136), Dobrava-Belgrade virus (DOBV, L33685), Andes virus (ANDV, AF291703), and Sin nombre virus (SNV, L25783) were codon-optimized and synthesized at GenScript (Nanjing, China). All target genes were cloned into the pCAGGS vector as previously indicated (Wang et al., 2022). HEK-293T was transfected with each hantavirus GPC vector using Hieff Trans Liposomal Transfection Reagent (Yeasen, China). At 24 h post-transfection (hpt), the cells were infected with pseudotyped VSV, which lacks the VSV-G gene but contains a GFP reporter ORF in its genome (VSVΔG) and bear VSV-G in trans (VSVΔG*G). The supernatants containing different hantavirus GPC pseudotyped VSV (pVSV) were harvested at 24 hpt, filtered, aliquoted, and stored at −80°C. pVSV bearing different hantaviral GPC was co-incubated with GRFT for 60 min at 37°C and added to 96-well microplates seeded with Vero E6 cells. At 12 to 24 hpi, the cells were visualized by a fluorescence microscope and processed as above indicated.

### *N*-Linked glycosylation mutants Hantaan virus glycoprotein precursor construction and pseudotyped vesicular stomatitis virus packing

Different primers used for HTNV GPC *N*-linked glycosylation mutants were synthesized at Sangon. Sequences are listed as follows: GPC-F: 5′-CAT TTT GGC AAA GAA TTC GCC ACC ATG GGC ATC TGG AAG TGG CTG GTG ATG G; GPC-R: 5′-GAG CCT CCA CCC CCG GTA CCA GAC TTC TTG TGC TTC CTC ACA GG; N235Q_Seg1-R: 5′-GCA GGT AGA TTC GAA GGA CTT CAC CTG CTC G; N235Q_Seg2-F: 5′-GAA GAA GTC CTT CGA ATC TAC CTG CCA GGA CAC CGA GAA CAA GGT GCA GG; N347Q_Seg1-R: 5′-CAG CTT GGG GAA CAG GCC TGG AGA GAA C; N347Q_Seg2-F: 5′-CTC TCC AGG CCT GTT CCC CAA GCT GCA CAC CAA CTG CGA CAA GTC CGC TAT C; N399Q_Seg1-R: 5′-GCC ACC TTC GCT GAA GGC CTC GCA AG; N399Q_Seg2-F: 5′-GGC CTT CAG CGA AGG TGG CAT CTT CCA GAT CAC CTC TCC TAT GTG CCT CGT G; N928Q_Seg1-R: 5′-GAA AGA CTG GAA GGA GTC GAT GGT GG; N928Q_Seg2-F: 5′-CAC CAT CGA CTC CTT CCA GTC TTT CCA GAC CTC CAC CAT GCA CTT CAC CGA C. Every single *N*-glycan mutation was conducted by amplifying fragment 1 and fragment 2 divided from the *N*-linked glycosylation site. The full length of HTNV GPC bearing corresponding N-Q mutation was obtained by overlap PCR. After single mutants were obtained, the double-site mutants and triple-site mutants were constructed based on single mutants. Each full-length HTNV GPC containing homologous recombination arms was inserted into the pCAGGS vector using NovoRec plus One Step PCR Cloning Kit (Novoprotein, Suzhou, China) following the manufacturer’s instructions. All constructs were sequence verified. These pseudotyped VSVs bearing the HTNV GPC *N*-linked glycosylation site mutants were packaged and submitted to GRFT inhibition testing as indicated above.

The structure of HTNV GPC was modeled based on the structure of the Andes virus (ANDV) (PDB: 6Y5F) using the SWISS-MODEL program (Meier et al., 2021). The location of each *N*-linked glycosylation site was indicated except for N399, which is truncated from the original structure.

### Statistical analysis

The statistical analysis was performed using GraphPad Prism software (La Jolla, CA, USA) using a two-tailed unpaired t-test. Data are presented as the means ± standard deviations (SDs) (n = 3 or otherwise indicated).

## Results

### Griffithsin inhibits rVSV-HTNV-GFP entry

**Figure 2:**
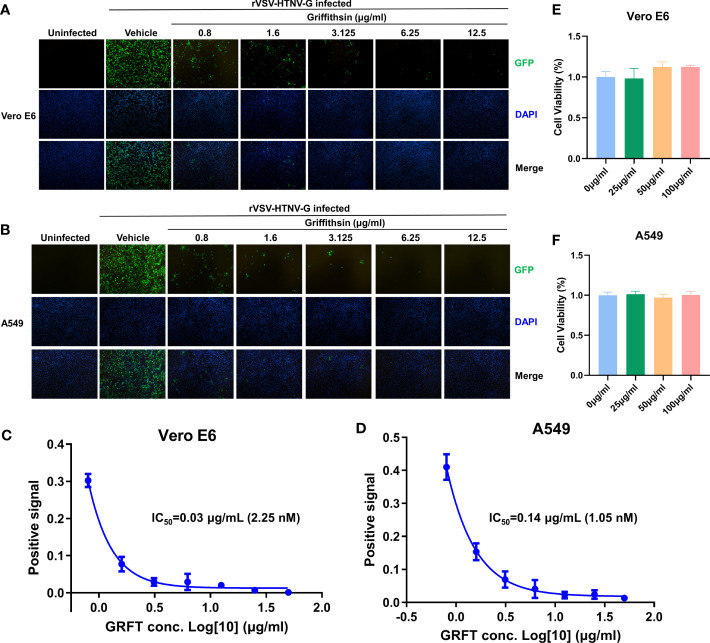
GRFT inhibits rVSV-HTNV-GFP entry. The recombinant vesicular stomatitis virus harboring HTNV glycoproteins (rVSV-HTNV-G) and GFP were treated with varying concentrations of GRFT for 1 h, and then the mixture was used to infect Vero-E6 **(A)** or A549 **(B)** cells at an MOI of 1. At 24 h post-infection, cell nuclei were stained with Hoechst 33258 and observed under an inverted fluorescence microscope. Green, GFP, indicates the replication of rVSV-HTNV-G; blue, cell nuclei. (C,D). The dose–response curve shows the quantification of GFP-positive Vero-E6 **(C)** or A549 **(D)** cell area after GRFT treatment of rVSV-HTNV-G (normalized with the positive control, the first point in the figure shows the first GRFT treatment concentration instead of positive control). **(E, F)** Cell viability was measured 72 h after drug administration using the Cell Counting Kit-8 (CCK8), and absorbance for Vero E6 **(E)** cells and A549 **(F)** cells was measured at 450 nm using the BioTek HT collaborative instrument. A450 levels were subtracted from blank well background, and untreated cells were set at 100% viability. Data shown in the graphs are presented as the mean ± SD and are representative of three independent experiments performed in hexaplicate. GRFT, griffithsin; rVSV, recombinant vesicular stomatitis virus; HTNV, Hantaan virus; GFP, green fluorescent protein; MOI, multiplicity of infection.

GRFT’s potency against anti-enveloped viruses, including SARS-CoV-2 viruses, was linked to its inhibition of viral binding to cell surface receptors (Cai et al., 2020). To determine whether GRFT inhibited HTNV entry, first, rVSV-HTNV-G was treated with different concentrations of GRFT for 1 h. Subsequently, the GRFT-treated rVSV-HTNV-G was used to infect Vero E6 cells (multiplicity of infection, MOI = 1), a cell line highly permissive for HTNV, with a mixture of GRFT and rVSV-HTNV-G. After 24 h, GFP-positive cells were quantified with an inverted fluorescence microscope. As shown in **Figure 2A**, GRFT inhibited rVSV-HTNV-G infection in a dose-dependent manner (**Figure 2A**). A comparable pattern was observed in A549 cells, but at higher concentrations (**Figure 2B**), the antiviral activity of GRFT was nearly five times lower in A549 than in Vero E6 cells (0.14 vs. 0.03 µg/ml) (**Figures 2C, D**). GRFT, however, did not affect cell viability even at 100 μg/ml (**Figures 2E, F**), emphasizing its safety. These results suggested that GRFT could inhibit rVSV-HTNV-G infection. As the glycoprotein on rVSV-HTNV-G and authentic HTNV were identical, we speculated that GRFT may inhibit the replication of the authentic virus as well.

### Griffithsin inhibits Hantaan virus infection

**Figure 3:**
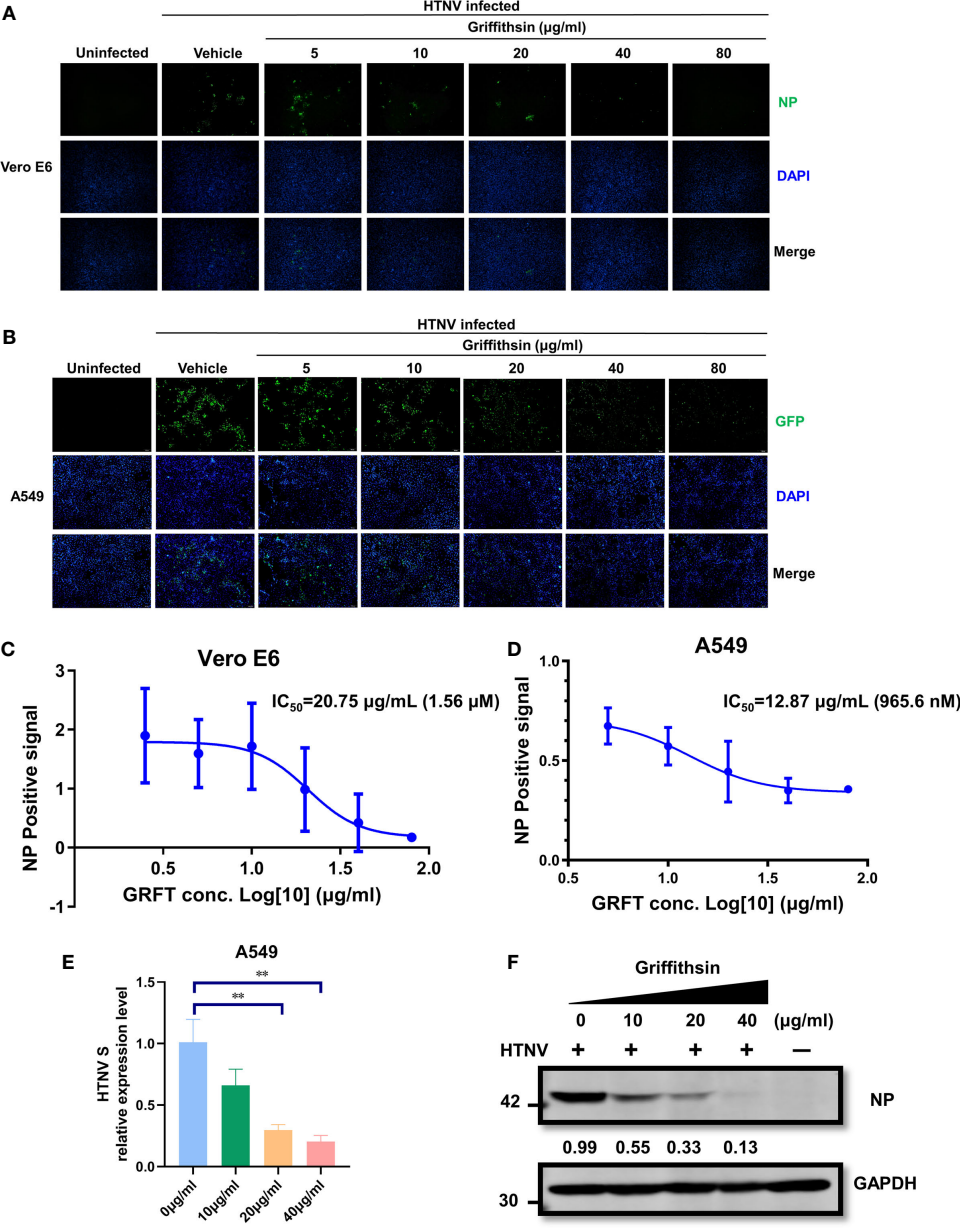
GRFT inhibits HTNV replication. **(A, B)** HTNV was treated with varying concentrations of GRFT for 1 h. The mixture was then used to infect Vero-E6 **(A)** or A549 **(B)** cells at an MOI of 1. At 72 h post-infection, the cells were fixed, permeabilized, and stained with a mouse monoclonal antibody 1A8 against HTNV nucleoprotein. Green, HTNV nucleoprotein; blue, cell nuclei. **(C, D)** The dose–response curve shows the quantitation of HTNV nucleoprotein-positive Vero-E6 **(C)** or A549 **(D)** cells after HTNV treatment with GRFT (% normalized to the vehicle-only control). **(E)** The HTNV S segment RNA level in A549 was detected and normalized to GAPDH. **(F)** The HTNV nucleoprotein in A549 cells was detected with an NP-specific antibody and normalized to the GAPDH level. Data shown in the graphs are presented as the mean ± SD and are representative of three independent experiments, performed in triplicate. **p < 0.01. GRFT, griffithsin; HTNV, Hantaan virus; MOI, multiplicity of infection.

The efficacy of GRFT as an antiviral agent against an authentic HTNV infection was further evaluated. HTNV was first pre-incubated at room temperature with increasing concentrations of GRFT for 1 h, and the mixture was then used to infect Vero E6 cells (MOI = 1). After 72 h, cells were fixed, permeabilized, and stained with nucleoprotein (NP)-specific monoclonal antibody 1A8 (Ye et al., 2019). Positive staining indicated HTNV infection, and stained cells were visualized and photographed. In Vero E6 cells (**Figure 3A**), GRFT inhibited HTNV infection, with IC_50_ of 20.75 μg/ml (1.56 μM) (**Figure 3C**). Similar results were observed in A549 cells (**Figure 3B**), with IC_50_ of 12.87 μg/ml (965.6 nM) (**Figure 3D**). Furthermore, compared with Vero E6 cells, RNA levels of HTNV (**Figure 3E**) and NP (**Figure 3F**) were significantly lower in HTNV-infected A549 cells treated with GRFT. Our results confirmed that GRFT inhibited the uptake of authentic HTNV by Vero E6 cells and A549 cells, indicating that it can prevent infections by multiple enveloped viruses.

### Griffithsin inhibits virus infection by binding *N*-linked high-mannose

**Figure 4:**
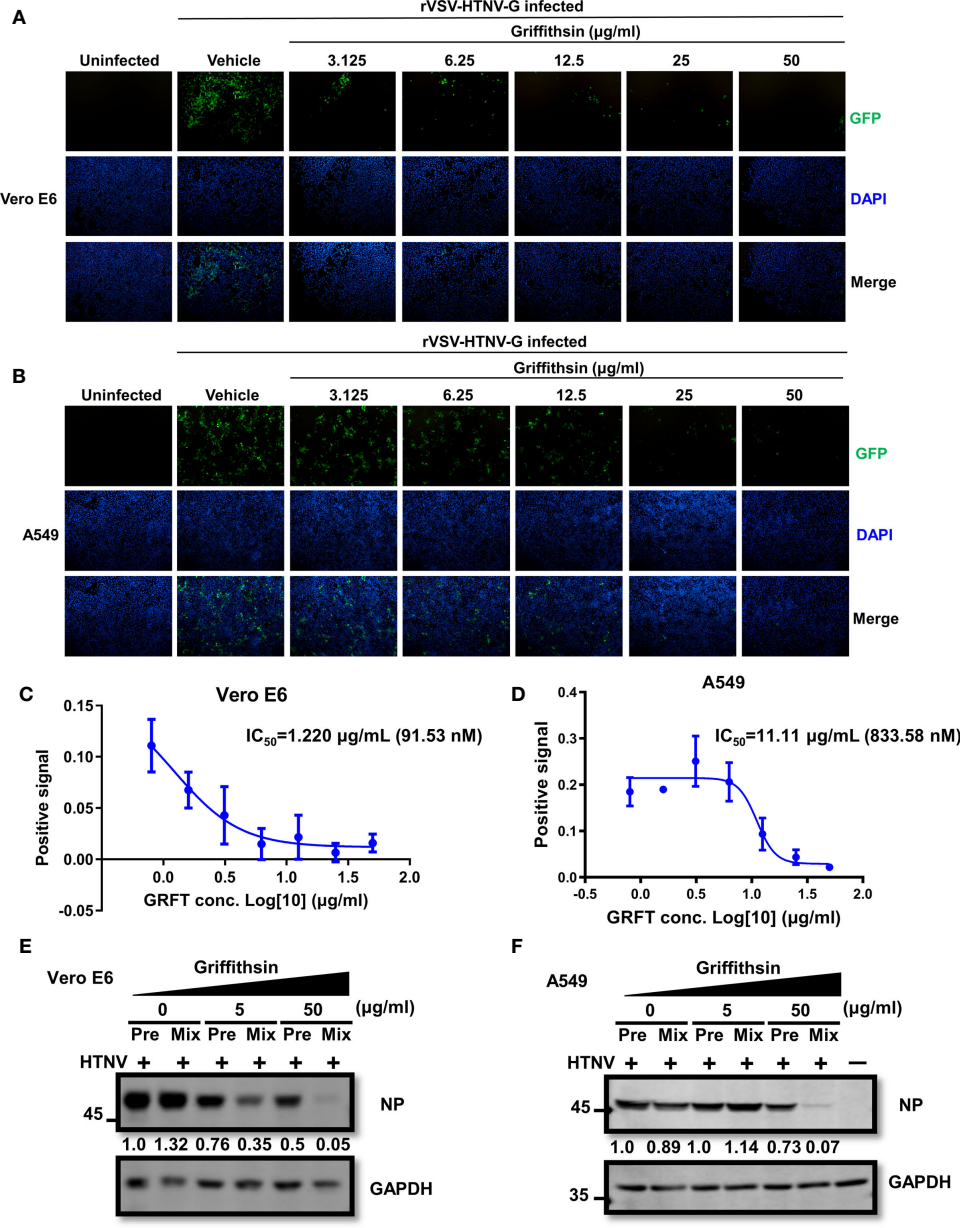
GRFT-pretreated cells reduce rVSV-HTNV-GFP and HTNV infection. **(A, B)** Vero-E6 cells **(A)** or A549 cells **(B)** were treated for 1 h with varying concentrations of GRFT and removed before being infected with rVSV-HTNV-G at an MOI of 1. At 24 h post-infection (hpi), the cell nuclei were stained with Hoechst 33258 and viewed under an inverted fluorescence microscope. Green, GFP, indicating the replication of rVSV-HTNV-G; blue, cell nuclei. **(C, D)** The quantitation of GFP-positive Vero-E6 **(C)** or A549 **(D)** cells after GRFT pretreatment of cells and infection with rVSV-HTNV-G (% normalized to the vehicle-only control) are shown in the dose–response curve. **(E, F)** The effects of GRFT antagonized HTNV infection in GRFT-pretreated cell group (Pre), or GRFT-treated virus group (Mix). Vero E6 **(E)** and A549 **(F)** cells were pretreated with 5 or 50 μg/ml of GRFT or vehicle and then infected with HTNV (MOI = 1), or equivalent HTNV was treated with 5 or 50 μg/ml of GRFT or vehicle for 1 h, and then the mixture was used to infect Vero E6 and A549 cells. At 72 hpi, the NP level within the cell was detected and normalized to GAPDH level. Data shown in the graphs are presented as the mean ± SDs and are representative of three independent experiments performed in triplicate. GRFT, griffithsin; rVSV, recombinant vesicular stomatitis virus; HTNV, Hantaan virus; MOI, multiplicity of infection; GFP, green fluorescent protein.

GRFT typically inhibits virus infection by binding *N*-linked high-mannose oligosaccharides on the viral glycoproteins. To further validate GRFT’s effectiveness against HTNV infection, A549 and Vero E6 cells were pretreated with varying concentrations of GRFT and incubated for 1 h at 37°C. After removing GRFT by washing the cells, GRFT-pretreated cells were infected with the rVSV-HTNV-G virus at an MOI of 1. Although these GRFT-pretreated cells inhibited rVSV-HTNV-G infection (**Figures 4A, B**), their efficacy was much lower than that of the GRFT-pretreated virus group. The IC_50_ in Vero E6 cells and A549 cells was 1.220 μg/ml (91.53 nM) and 11.11 μg/ml (833.58 nM), respectively (**Figures 4C, D**). Thus, GRFT binds to cells to prevent viral entry, although its binding is not efficient. Subsequently, authentic HTNV cells treated with GRFT and Vero E6 cells pretreated with GRFT were infected with HTNV at an MOI of 1 for 72 h. The cells were subjected to the Western blotting assay, which revealed a significant reduction in NP expression levels in GRFT-treated viral groups compared with cells pretreated with high concentrations of GRFT (**Figures 4E, F**).

### Hantaan virus neutralization by griffithsin is dependent on carbohydrate-binding sites on viral glycoprotein

**Figure 5:**
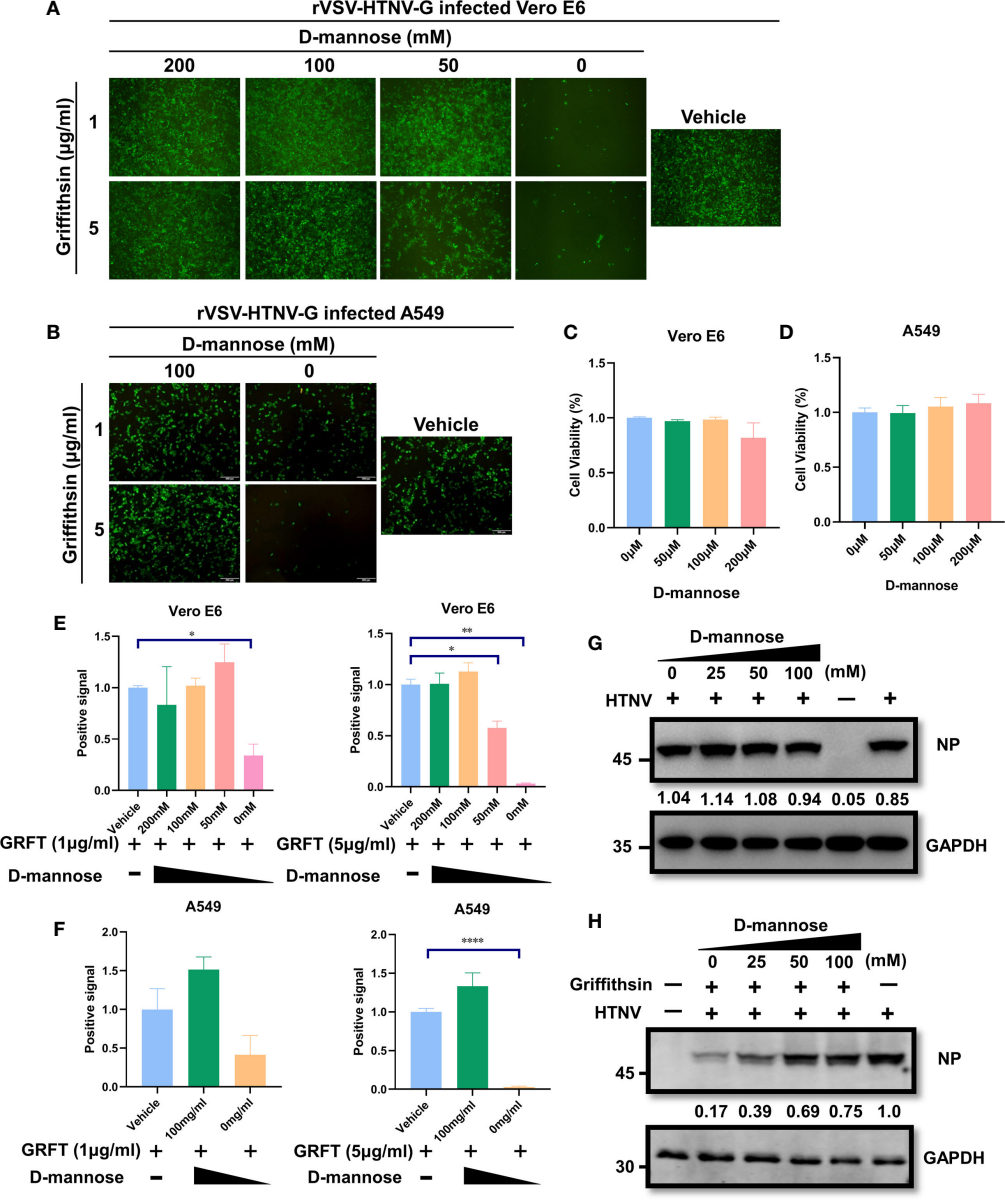
The antiviral effect of GRFT against HTNV depends on the carbohydrate-binding sites on the viral glycoprotein. **(A)** 5 or 1 μg/ml of GRFT was pre-incubated with 0, 50, 100, or 200 mM of d-mannose for 1 h and then incubated rVSV-HTNV-GFP for an additional 1 h. The triple mixture was used to infect Vero E6 cells (MOI = 5) and viewed at 12 hpi. Vehicle refers to the treatment group infected with a virus only, without GRFT and d-mannose. **(B)** A549 cells were treated similarly to Vero E6 cells but using only 100 mM of d-mannose. **(C, D)** Cell viability was assayed using CCK8, with absorbance **(A)** at 450 nm. Vero E6 **(C)** or A549 **(D)** cells were treated with increasing concentrations of d-mannose, 72 h post-treatment. **(E, F)** The dose-dependent d-mannose treatment dampened the inhibitory effect of GRFT on Vero-E6 **(E)** or A549 **(F)** cells. Data shown in the graphs are presented as the mean ± SDs and are representative of three independent experiments, performed in triplicate. *p < 0.05, **p < 0.01, ****p < 0.0001. **(G)** d-Mannose treatment did not affect HTNV infection. HTNV was pretreated at different concentrations of d-mannose for 1 h, and then the mixture was used to infect Vero E6 cells, at 72 hpi, and the NP level within the cell was detected and normalized to GAPDH level. **(H)** GRFT (50 μg/ml) was pretreated with different concentrations of d-mannose for 1 h and then mixed with HTNV. The triple mixture was used to infect Vero E6 cells, and the NP level was detected at 72 hpi and normalized to GAPDH level. GRFT, griffithsin; HTNV, Hantaan virus; rVSV, recombinant vesicular stomatitis virus; MOI, multiplicity of infection; CCK8, Cell Counting Kit-8.

Since GRFT functions by binding high-mannose oligosaccharides on viral glycoproteins, pre-occupation of carbohydrate-binding sites with mannose oligosaccharides may diminish its antiviral properties. To test this hypothesis, GRFT was treated with varying concentrations of d-mannose for 1 h at room temperature, followed by incubation with rVSV-HTNV-G for 1 h. The ternary mixture was then used to infect Vero E6 cells. As illustrated in **Figures 5A, E**, the inhibitory activity of GRFT against rVSV-HTNV-G infection was significantly attenuated when pretreated with a high concentration of mannose. This suggested that GRFT inhibits rVSV-HTNV-G infection by targeting the glycosylation sites on the GPC, which were saturated by excess mannose levels. Pretreatment with high concentrations of mannose caused loss of antiviral activity of GRFT against rVSV-HTNV-G (**Figures 5B, F**). In addition, all d-mannose tests showed no evidence of cytotoxicity to Vero E6 and A549 cells (**Figures 5C, D**). Moreover, d-mannose treatment alone had no effect on the authentic HTNV infection (**Figure 5G**). In contrast, a high concentration of d-mannose dampened the inhibitory effect of GRFT against infections (**Figure 5H**). In conclusion, carbohydrate binding sites on GRFT are critical to their ability to inhibit HTNV infection.

### *N*-Linked glycosylation site on Hantaan virus glycoprotein precursor is the target of griffithsin

GRFT binds oligosaccharides through their carbohydrate binding sites. HTNV GPC contains five *N*-linked glycosylation sites, namely, N134, N235, N347, N399, and N928 (**Figure 1**). The N928 site is located in Gc, whereas the other four sites are located in Gn. To investigate the effect of *N*-linked glycosylation sites on the inhibitory activity of GRFT against HTNV infection, we constructed single N to Q mutants with missing glycosylation sites but with similar side chains. With the exception of N134, previous studies have suggested that the first *N*-linked glycosylation site is essential for the folding and stability of Gn glycoprotein (Shi and Elliott, 2004). Additionally, double mutants (N347Q-928Q and N399Q-N928Q) and triple mutants (N347Q-N399Q-N928Q) were constructed, with the exception of N235-399Q, which also results in Gn misfolding; moreover, the N235 site is missing in ANDV GPC (Shi and Elliott, 2004; Serris et al., 2020). That is, four single mutants, two double mutants, and one triple mutant were subjugated for pseudotyped VSV packaging and used to evaluate the inhibitory effects of GRFT.

**Figure 6:**
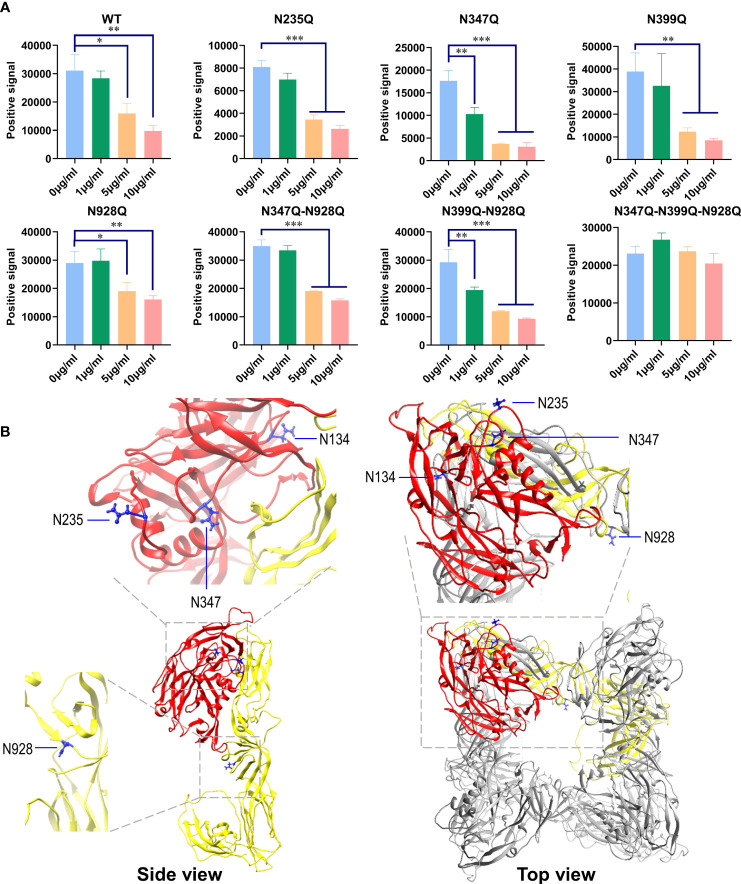
*N*-Linked glycosylation site on HTNV GPC is responsible for GRFT exerting HTNV inhibition effects. **(A)** HTNV GPC harboring different *N*-linked glycosylation site mutations was constructed using overlapping PCR. pVSV pseudotyped with these mutants was generated, and the infectious dose was normalized. Equal amounts of different pVSV-HTNV were pretreated with different concentrations of GRFT for 1 h and used to infect A549 cells. At 12 hpi, the GFP positive field was imaged and calculated. Data shown in the graphs are presented as the mean ± SDs and are representative of three independent experiments, performed in triplicate. *p < 0.05, **p < 0.01, ***p < 0.001. **(B)** The structure of the envelope glycoprotein perfusion complex of HTNV was modeled using the SWISS-MODEL program based on the envelope glycoprotein perfusion complex of the Andes virus (PDB: 6Y5F) and visualized using Chimera. Gn is colored red and Gc is yellow. The *N*-linked glycosylation residues, N134, N235, N347, and N928, are highlighted in blue. Except for N399, the corresponding ANDV N402 is missing in the original structure. HTNV, Hantaan virus; GPC, glycoprotein precursor; GRFT, griffithsin; pVSV, pseudotyped vesicular stomatitis virus.

As shown in **Figure 6A**, GRFT inhibited single mutants N235Q, N347Q, N399Q, and N928Q and double mutants N347Q-N928Q and N399Q-N928Q *via* VSV pseudovirus transduction. However, the triple mutant N347Q-N399Q-N928Q virtually eliminated the inhibitory effects of GRFT. These results indicated that the redundancy between these *N*-glycans was responsible for the inhibitory effects of GRFT. GRFT was, however, unable to inhibit HTNV infection when its main *N*-glycan sites at residues N347, N399, and N928 were removed. Furthermore, the structure model revealed that some of these glycans were distributed on the surface of GPC (**Figure 6B**). Therefore, binding of these glycans to GRFT created steric hindrance, preventing HTNV GPC from binding to the cellular receptor. Overall, the *N*-linked glycosylation site on HTNV GPC is responsible for GRFT’s inhibitory effect on HTNV infection.

### *In vivo* evaluation of antiviral activity of griffithsin against Hantaan virus infection

**Figure 7:**
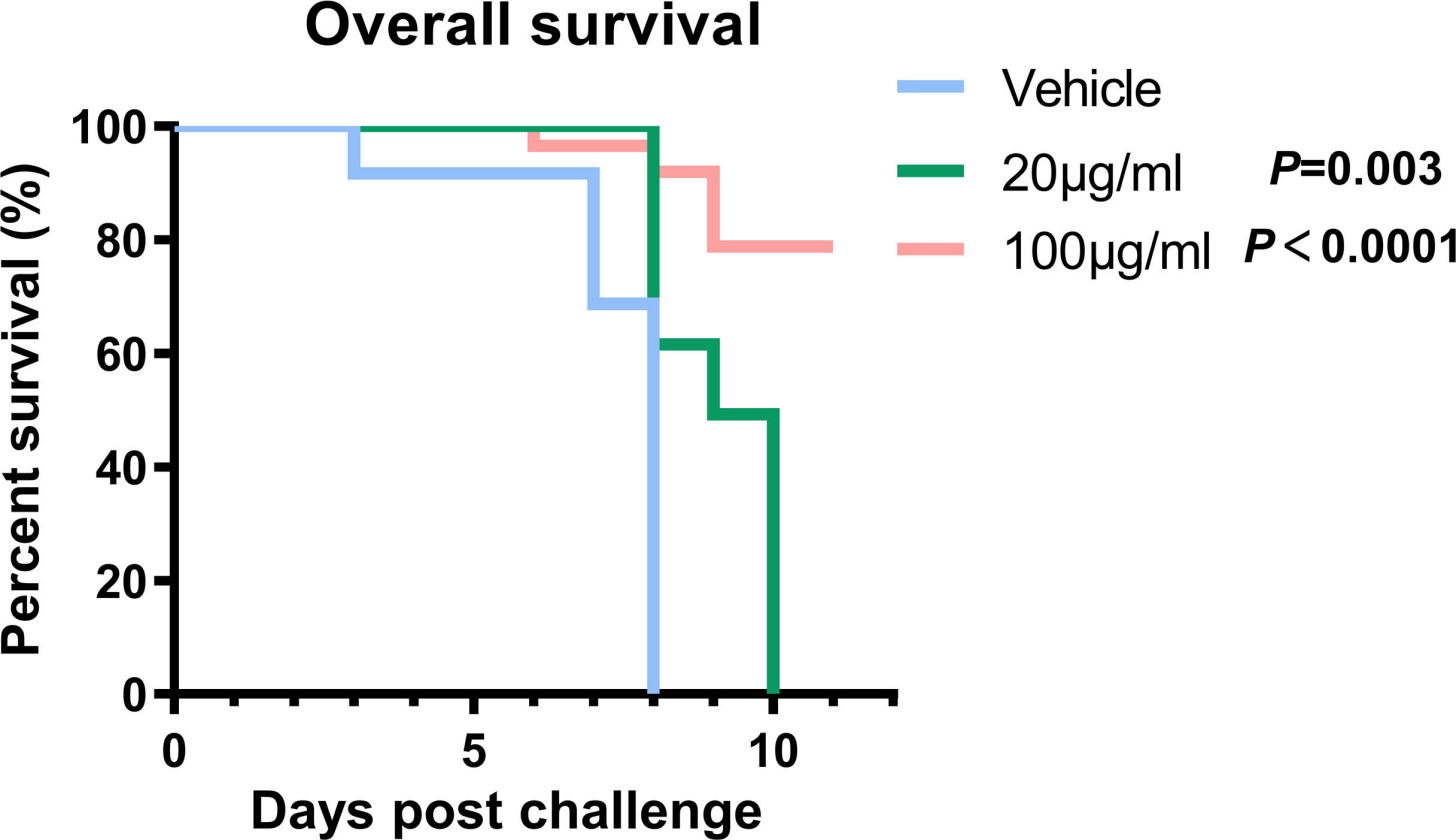
GRFT exerts HTNV inhibition effects *in vivo*. Briefly, 10 LD_50_ of HTNV was pretreated with 20 μg/ml (0.4 mg/kg) or 100 μg/ml (2 mg/kg) of GRFT for 1 h, and the mixture was used to inoculate suckling mice intracranially. Among them, 17 mice were in the control group (DMEM-only vehicle), 11 in 20 μg/ml, and 14 in 100 μg/ml. Mice were observed for 10 days, and the survival of the mice was recorded every day, presented as Kaplan–Meier survival curves, and analyzed using the log-rank test. GRFT, griffithsin; HTNV, Hantaan virus; DMEM, Dulbecco’s modified Eagle’s medium.

To further validate GRFT’s ability to treat HTNV infection *in vivo*, GRFT and HTNV were co-delivered intracranially to suckling mice. Briefly, three litters of 3-day-old suckling mice were injected with HTNV (10 LD_50_), which had been pretreated with increasing concentrations of GRFT as shown in **Figure 7**. When mice were treated with GRFT at a low concentration (20 μg/ml, 0.4 mg/kg), death was delayed, but at a higher concentration (100 μg/ml, 2 mg/kg), an 80% survival rate was achieved. In comparison, all control mice died between days 4 and 8 post-infection (**Figure 7**). Thus, GRFT treatment significantly improved the survival rate compared with vehicle treatment. Our results indicated that GRFT also inhibits the infection of HTNV *in vivo*.

### Griffithsin exerts pan-hantaviral inhibition effects

**Figure 8:**
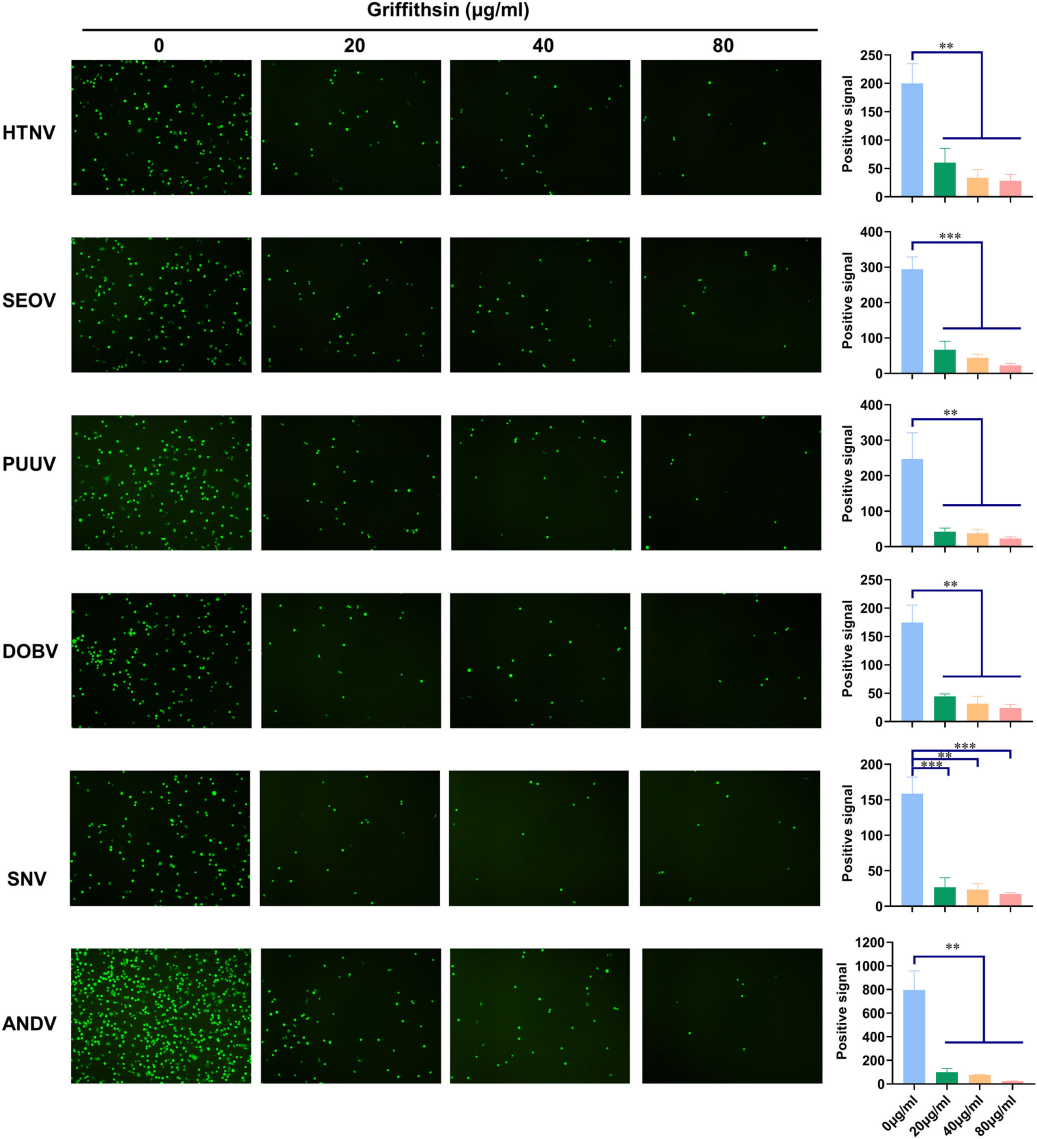
GRFT exerts pan-hantaviral inhibition effects. pVSV-based pseudotyped virus-bearing glycoproteins of HTNV, SEOV, PUUV, DOBV, SNV, and ANDV were prepared as indicated. pVSV of different hantaviral glycoproteins was pretreated with 20, 40, or 80 μg/ml of GRFT, and the mixture was used to inoculate A549 cells. Fluorescence was observed 24 h post-infection. Data shown in the graphs are presented as the mean ± SDs and are representative of three independent experiments, performed in triplicate. **p < 0.01, ***p < 0.001. GRFT, griffithsin; pVSV, pseudotyped vesicular stomatitis virus; HTNV, Hantaan virus.

A previous study found that GRFT inhibited New World hantaviruses, including ANDV and SNV, which are responsible for Hantavirus Pulmonary Syndrome (HPS) with mortality rates of up to 45% (Shrivastava-Ranjan et al., 2020). To evaluate the inhibitory spectrum of GRFT against hantaviruses, we generated pVSV with HFRS-causing hantaviral envelope glycoproteins SEOV, PUUV, and DOBV, as well as HPS-causing ANDV and SNV. In addition to inhibiting HTNV, ANDV, and SNV, GRFT also inhibited the infection of pVSV-SEOV, pVSV-PUUV, and pVSV-DOBV in a dose-dependent manner (**Figure 8**), demonstrating that the inhibitory effect of GRFT on hantavirus infection is independent of strain.

## Discussion

The lack of effective treatments for hemorrhagic fever with renal syndrome in China has increased the health burden of this condition in endemic regions, especially rural areas (Jiang et al., 2017). The main pathogen in these areas is HTNV, which has a higher mortality rate than SEOV. Our *in vitro* and *in vivo* experiments confirmed the potential efficacy of GRFT for the treatment of HTNV infection.

GRFT protein was isolated from *Griffithsin* sp., red marine algae found in the National Cancer Institute’s Natural Products Repository. GRFT folds into a stable domain-swapped dimer containing six carbohydrate-binding sites, which are responsible for its antiviral activity against various enveloped viruses, including herpes virus (Nixon et al., 2013; Tyo et al., 2020), Nipah virus (Lo et al., 2020), Japanese encephalitis virus (Ishag et al., 2013), hepatitis C virus (Meuleman et al., 2011), coronavirus (Millet et al., 2016; Li et al., 2019; Cai et al., 2020; Alsaidi et al., 2021), and HIV (Mori et al., 2005), demonstrating GRFT’s effective and broad-spectrum viral entry inhibition.

Furthermore, GRFT has been reported to be expressed in multiple organisms, including prokaryotes (*Escherichia coli*) and plants (*Nicotiana benthamiana* and *Oryza sativa* endosperm) (Giomarelli et al., 2006; Fuqua et al., 2015; Alam et al., 2018; Decker et al., 2020; Corman et al., 2020). The large-scale production and ease of purification make GRFT suitable for clinical application. A phase I clinical trial of GRFT against HIV in healthy volunteers also demonstrated its safety in humans and efficacy as a topical microbicide (Zeitlin et al., 2009).

HTNV contains four possible sites for *N*-linked glycosylation on Gn and one on Gc (**Figure 1**) (Schmaljohn et al., 1987; Antic et al., 1992; Shi and Elliott, 2004). Four dimers of Gn and Gc form the core building blocks of the viral spike, which covers the entire surface of the virion (Hepojoki et al., 2010; Huiskonen et al., 2010; Meier et al., 2021). This suggests that GRFT may effectively inhibit HTNV infection. Using rVSV expressing HTNV GPC and a GFP reporter, we observed a significant reduction in the infection level of rVSV-HTNV-G in Vero E6 and A549 cells. These results were further confirmed experimentally with authentic HTNV.

Meanwhile, the infection of A549 cells with live HTNV is significantly less responsive to GRFT. A previous study reported that HTNV may utilize macropinocytosis to infect human airway epithelial cells, such as A549 (Torriani et al., 2019). This kind of entry is likely mediated by receptor T-cell immunoglobulin and mucin (Mayor et al., 2020) and occurs independent of the endocytosis pathway, potentially contributing to the attenuated inhibitory effect of GRFT on HTNV infection in A549 cells.

Recent structural studies suggest that two *N*-linked glycans (N399 and N928) buried within the spike in HTNV (Serris et al., 2020) may not shield the virus from host immunity or act as an attachment factor like *N*-glycans observed in HIV. Because of this feature, *N*-linked glycans of hantaviruses remain high-mannose oligosaccharides, which are the preferred target of GRFT.

Considering that GRFT can recognize and bind mannose, pretreatment of GRFT with mannose suppressed the inhibitory effect of GRFT on viruses (**Figure 5**), confirming that GRFT’s HTNV inhibition is a carbohydrate-binding site-dependent phenomenon. Therefore, GRFT binds to and cross-links glycans on the virion surface, preventing its structure from shifting or accessing the cell surface receptor. Several pseudotyped HTNV GPC VSVs bearing mutants at different *N*-linked sites were generated to verify this hypothesis. It is noteworthy that GRFT’s inhibitory effect on pVSV infection was nearly eliminated in a triple mutant N347Q-N399Q-N928Q (**Figure 6A**), but not in single or double mutants, suggesting the possibility that one GPC can bind multiple GRFT molecules. In the modeled structure (**Figure 6B**), N134 is less exposed compared to other glycosylation sites. This may explain that in the triple mutant, GRFT is unable to inhibit the HTNV-GP pVSV glycosylated at N134. Additionally, the glycan at N134 may not sterically fit properly into the GRFT pocket and thus contribute little to GRFT inhibition of HTNV infection. However, this still needs further investigation.

Therefore, if one *N*-glycan is missing, GRFT can still neutralize the protein sufficiently, which suggests a high resistance barrier for HTNV to overcome the inhibitory effect of GRFT.

Considering that cells pretreated with GRFT could reduce HTNV infection but with lower efficiency compared with virus pretreated with GRFT (**Figure 4**), it is reasonable to speculate that GRFT’s binding to viral cells does not completely prevent the virus from interacting with cellular receptors to enter the cells. A similar phenomenon was observed in other viruses, including SARS-CoV-2 (Cai et al., 2020). Although GRFT reached a saturated antiviral effect at higher concentrations, more research is needed to determine the precise mechanism by which GRFT inhibits HTNV infection.

Studies have also demonstrated that GRFT can inhibit viral infections in mouse models (O'Keefe et al., 2010; Ishag et al., 2013; Takebe et al., 2013; Barton et al., 2014). However, rodents are the natural hosts of the HFRS-causing hantavirus. Therefore, infection with HTNV or SEOV in adult mice only results in transient infections that soon resolve without complications. Since adult mice infected with HTNV do not display obvious symptoms, the suckling mouse model is widely used as an alternative model for testing neutralizing antibodies and antiviral drugs. GRFT treatment provided partial protection against HTNV infection in suckling mice infected with HTNV (**Figure 7**), indicating the potential use of GRFT as a treatment against HTNV *in vivo*. Also, a relatively long pretreatment of GRFT with the virus was needed for GRFT to exhibit efficient antiviral activity. This implies that GRFT acts like neutralization antibodies and that several injections may be needed in a proper animal model, e.g., non-human primates.

Additionally, there are two types of hantaviruses based on geographic distribution and type of disease caused: HFRS and HPS. However, these hantaviruses have glycosylated GPCs, and GRFT shows a broad spectrum anti-hantaviral activity. As a result, GRFT may be developed as a pan-antihantaviral agent.

Our study demonstrates that GRFT inhibits HTNV infection both *in vitro* and *in vivo*. Its ease of production and high safety make it an attractive candidate for further development as an anti-HTNV drug.

## Data availability statement

The original contributions presented in the study are included in the article. Further inquiries can be directed to the corresponding authors.

## Ethics statement

The animal study was reviewed and approved by Animal Care and Use Committee OF Airforce Medical University.

## Author contributions

Conceptualization, WY, LL, and YC; methodology, YZ, NZ, YC, HZ, WY; validation, NZ, JQL, WL; formal analysis, NZ and WY; investigation, YZ, NZ, JQL, CY and WY; resources, JL, YMD, LZ, YFL, WYL, YW, HL, CY, LQZ, YXL, LZ, LFC, YMD and LL; writing, original draft preparation, WY and NZ; writing, review and editing, YC, YFL, FZ.; supervision, YJW, FZ; project administration, ZX, YJW, YFL, FZ and WY; funding acquisition, ZX, FZ and WY. All authors have read and agreed to the published version of the manuscript.
